# Therapeutic Drug Monitoring and Point-of-Care Technologies: Opportunities and Current Challenges

**DOI:** 10.1097/FTD.0000000000001384

**Published:** 2025-10-07

**Authors:** Sandro Carrara, Nicolas Widmer, Francesca Rodino, Lin Du, Myriam Briki, Laurent A. Decosterd, Catia Marzolini, Thierry Buclin, Yann Thoma, Monia Guidi

**Affiliations:** * Bio/CMOS Interfaces Laboratory, École Polytechnique Fédérale de Lausanne (EPFL), Neuchâtel, Switzerland;; † Service of Clinical Pharmacology, Department of Medicine and Pathology, Lausanne University Hospital (CHUV) and University of Lausanne (UNIL), Lausanne, Switzerland;; ‡ Institute of Pharmaceutical Sciences of Western Switzerland (ISPSO), University of Geneva, University of Lausanne, Geneva, Switzerland;; § Pharmacy of the Eastern Vaud Hospitals (PHEL), Rennaz, Switzerland;; ¶ School of Engineering and Management Vaud, University of Applied Sciences and Arts Western Switzerland (HES-SO), Yverdon-les-Bains, Switzerland;; ║ Division of Infectious Diseases and Hospital Epidemiology, University Hospital Basel and University of Basel, Basel, Switzerland;; ** Department of Molecular and Clinical Pharmacology, Institute of Translational Medicine; University of Liverpool, Liverpool, United Kingdom; and; †† Centre for Research and Innovation in Clinical Pharmaceutical Sciences, Lausanne University Hospital (CHUV) and University of Lausanne (UNIL), Lausanne, Switzerland.

**Keywords:** point-of-care systems, bedside technology, pharmacokinetics, machine learning, model-informed precision dosing

## Abstract

**Background:**

This review re-evaluates therapeutic drug monitoring (TDM) by comparing the current analytical and subsequent clinical interpretation capabilities of hospital or community medical laboratories with the emerging potential of point-of-care (POC) devices, which could become increasingly utilized in hospital wards, day-hospital units, and outpatient clinic settings.

**Methods:**

A narrative review was conducted to identify publications that best illustrate the current trends in the development of POC TDM.

**Results:**

The latest scientific and technical literature indicates that POC devices for determining drug concentrations in clinical samples are approaching the market. Several technologies are now available to develop portable sensors capable of rapidly returning concentration measurements. Interfacing these methods with artificial intelligence-based pattern recognition may enhance the identification and quantification of drugs. However, once the drug concentration is accurately measured using a portable device, dosage adjustments require consideration of the drug's pharmacokinetics and the patient's characteristics. This is accounted for in the mathematical approaches underlying model-informed precision dosing, which consider inter- and intra-individual variability and provide recommendations for treatment adjustments. These complexities necessitate the use of digital technologies, including graphical interfaces, machine learning approaches, and secure connectivity, to enhance the application of TDM in clinical practice.

**Conclusions:**

Promising emerging technologies have considerable potential to expand TDM to cover a wide range of drugs, making precision medicine accessible to many patients.

## INTRODUCTION

The dynamics of pharmacotherapy development significantly differ before and after drug approval. Indeed, on the one hand, premarketing drug development has evolved efficiently: (1) novel routes to drug discovery use combinatorial chemistry, in silico molecular docking, virtual screening, deep learning, and artificial intelligence (AI); (2) fundamental pharmacology dissects the mechanisms underlying the action of therapeutic agents from the molecular level up to systems pharmacology; (3) clinical pharmacometrics enable the analysis, modeling, and simulation of pharmacokinetic (PK) and pharmacodynamic (PD) characteristics of drugs to best shape the therapeutic efficacy and tolerability. Collectively integrated into Model-Informed Drug Development (MIDD), these approaches have surely transformed pharmacotherapy from discovery to regulatory approval.^1,2^ On the other hand, following commercialization, the individualization of drug dosage in real-world patients has been relatively neglected.^3–5^ However, the same technological and methodological advances supporting MIDD have been advocated for tailoring on-market treatments to individual patients in Model-Informed Precision Dosing (MIPD), an integrated approach.^6,7^ For a significant portion of drugs, MIPD can be limited to consider specific a priori criteria that influence the dosage for ensuring efficacy without compromising safety (eg, body weight or body surface area, age, sex, renal function, disease severity score, pharmacogenetic markers, and interacting comedications). Meanwhile, for other treatments, the therapeutic response itself can be measured, offering a preferred MIPD criterion based on clinical feedback.^8^ Still, several drugs require dosage individualization based on an a posteriori assessment of circulating concentration exposure under initial dosing, that is, therapeutic drug monitoring (TDM), a privileged marker on which to base MIPD. Precision dosing is no less critical than personalized drug choice in *precision medicine*, defined as “the provision of the right drug at the right dose to the right patient.”^9,10^

TDM has long been restricted to a handful of classical drugs currently measured in most clinical laboratories (eg, lithium, digoxin, phenytoin, aminoglycosides, vancomycin, cyclosporine, and tacrolimus). However, many more drugs admittedly require TDM-based dosing adjustment, considering the classical criteria that define suitable TDM candidates.^11^ Nevertheless, the desirable development and implementation of hundreds of TDM tests seem to remain slow and haphazard, if not elusive, and continue to face obstacles.^12^ Clinical trials involving concentration adjustments are largely disregarded by industry sponsors with concerns regarding the unappealing flavor of TDM prospects for drug marketing. The question of who should be responsible for developing a novel TDM remains unanswered.^13^ But above all, from a practical point of view, both the analytical procedures and clinical interpretations involved in TDM remain complex and demanding for healthcare providers. Consequently, even for highly effective new drugs, insufficient individualization of dosing is likely to deprive a significant proportion of patients of optimal treatment efficacy and tolerability.

Therefore, it is reasonable to expect that TDM will only expand to the target level when it is based on straightforward, quick, and cost-effective procedures. The forthcoming development of miniaturized medical devices capable of measuring drug concentrations directly at the point of care (POC), combined with high-performance MIPD calculation tools, can advance TDM, which could become familiar to practitioners as electrocardiography or portable ultrasound.

Over the last few decades, several approaches have been proposed to develop POC technologies for TDM, the earliest of which, developed in the 1980s, were relatively large devices proposed for physician office laboratories.^14^ As discussed in Section 4, several technical opportunities now exist for developing portable sensors that can detect therapeutic compounds in the patients' blood, each of which may yield an appropriate POC technology that is typically easy to use with limited fabrication costs. Most currently proposed sensing technologies are based on either electrochemical or optical sensors. In both cases, if the therapeutic compound is measured within the correct concentration range, the POC technology then becomes possible by coupling the related sensor with a proper electronic system, typically a frontend to drive and read the sensor, coupled with a backend that processes the data and returns concentration values. Data can then be visualized through the device's user interface or easily transmitted wirelessly to portable user devices, such as microcomputers, tablets, smartphones, and smartwatches.^15^

However, the introduction of portable devices in clinics for measuring active pharmaceutical substances during therapy has been slow so far, for reasons that extend beyond technological capabilities. The reason often alleged relies on a certain inertia that remains pervasive in clinical practice: physicians are reluctant to adopt new routines until backed by strong recommendations based on convincing evidence and endorsed by authoritative experts, third-party payers are rigorous in their criteria for TDM cost coverage, pharmaceutical companies are usually not enthusiastic about TDM, and public funders are reluctant to invest in drug-related research. Thus, TDM clinical trials struggle to find financial support, hence completing a vicious circle.^16^

Another reason is the difficulty of the approaches proposed to deliver easy-to-understand results that are practical in real-life practice for prescribers.^16^ The difference between the typical outcome of an analytical measure and its understandable use in clinical practice can be considered the primary reason for the gap existing about TDM between the scientific and the clinical communities.^17^ Section 6 examines how efforts to digitize the therapeutic decision may address the latter problem.

This review discusses the current state of these advances and the avenues they open for POC TDM. It begins by overseeing the general context of prescribers and patients' needs, which should lead to improvements in this field, describes recent developments and prospects for miniaturized bioelectronic drug sensors, and examines the evolution of MIPD software tools, an essential element for the medical use of drug concentration measurements in terms of precision dosing. Finally, these developments are discussed within the broader context of digital healthcare integration, concluding with plausible prospects.

## METHODS

Owing to the high multidisciplinarity of this topic and the extensive discussions involved, this review was conducted by a diverse group of authors with backgrounds in biomedical engineering, pharmacy, medicine, computer science, medical biology, and pharmacometrics modeling and simulation. The group collected publications that best illustrated the current trends and future developments in POC TDM. Rather than covering extensive literature, which would have been overwhelming, the goal was to select the most relevant articles that best illustrated our line of reasoning regarding the prerequisites, enabling factors, and current challenges for this biotechnological development.

## CURRENT AND UPCOMING NEEDS

The primary requirement of healthcare professionals and patients in terms of precision dosing is to extend TDM to a large number of drugs throughout the healthcare system, which is far greater than is currently the case. Many scholars have suggested TDM to be widely offered for most, if not all, anti-infective drugs used in critical care units,^18^ both classical and recent antitubercular agents,^19^ many antiretrovirals,^20^ antifungals,^21^ antiepileptics,^22^ traditional anticancer chemotherapies,^23^ small-molecule targeted anticancer agents,^24^ psychotropic drugs,^25^ and biologicals.^26^ Furthermore, new drugs are being considered as likely candidates, such as long-acting antistaphylococcal glycopeptides,^27^ chloride channel modulators used in cystic fibrosis,^28^ or even engineered CAR-T cells infused against malignancies.^29^ Even if laboratory machinery evolves to cope with large numbers of samples, the generalization of TDM to hundreds of different drugs will make today's approach to dosage adjustment unsustainable, as it is too time-consuming, human-skill-intensive, and costly. The number of candidate drugs for TDM no longer allows prescribers or clinical pharmacists and pharmacologists to know their pharmacokinetics and exposure targets by heart, and or to adapt their dosage accordingly. Technological support will inevitably be needed not only for the analytical side of TDM, but also, and perhaps above all, for its clinical PK and PD side.

The second essential need for prescribers is to shorten the feedback loop, which begins with drug prescription, passes through biological sampling, laboratory analysis, MIPD-supported interpretation, and ends with dosage adjustment. This process currently takes several hours in an efficient hospital and several days in outpatient practice. Clinical pharmacology and chemistry laboratories utilize large, high-performance analytical instruments, typically working on a series of samples that often require preparation and processing in batches. The clinical interpretation of the measurement results remains largely conducted by specialists on an empirical basis. Although MIPD software exists and is available, it is rarely integrated with laboratory information management systems (LIMSs) and/or electronic health records (EHRs); therefore, implementation often requires manual data entry into the application. Transmission of dosage adjustment recommendations to prescribers also requires time. The duration of the entire process compromises the utility of TDM in certain situations. For example, 5-fluorouracil is a classical anticancer chemotherapy known to benefit from TDM and is usually administered in monthly 24-h or 48-h infusions. In rare centers offering TDM of 5-fluorouracil, sample measurements are performed to modify the dosage in the next cycle, that is, 1 month later, when the patient's condition might have changed. Rapid POC TDM might enable modification of the infusion rate or duration in the same cycle as the sample.^30^

The third relevant need for healthcare providers and patients is for TDM to be connected and integrated into the digitization of health care, along with other key components of patient management. Compared with other laboratory tests, TDM produces results that are nontrivial to interpret and require knowledge of the current dosage, sampling schedules, and influential clinical variables. Moreover, MIPD requires reference population PK models and sophisticated calculations, which must be smartly summarized and rendered to busy clinicians who are rarely equipped with extensive pharmacokinetic skills. Therefore, numerical procedures supporting the interpretation of results must be integrated into efficient clinical decision support systems (CDSS) to be widely adopted by professionals.

Globally, patient EHRs tend to become increasingly connected with extensive knowledge bases, providing the best evidence to support diagnostic and therapeutic decisions (ie, “evidence-based medicine”). These connections should work bi-directionally, allowing each patient's story to feed into vast collections of data that enable medical knowledge to be updated on the fly, a process designated as “medicine-based evidence.”^31^ Regarding specifically TDM and MIPD, such data acquisition is of prime importance to update and expand reference population PK/PD models. Regarding data exchange, the importance of managing security and confidentiality should not be overlooked.

Finally, the convenience aspects of TDM for patients deserve consideration. Traditional TDM requires sampling of the patient's blood precisely at a concentration trough, that is, at the end of a dosing interval, at steady state under a constant therapeutic regimen, before being sent to a medical laboratory. Current MIPD approaches allow sampling to be carried out at random times after the last dose, under a varying dosing schedule, provided the corresponding information is taken into account in calculations.^32^ Rather than requiring the classic blood tube taken by venipuncture and centrifuged within 30 minutes, the TDM of the future could accommodate less invasive sampling, such as a single drop of blood, saliva, or sweat, thus allowing a measurement to be made directly at the patient's side.^33^

## POINT-OF-CARE TECHNOLOGIES: LOOMING OPPORTUNITIES

The above discussion highlights the importance of introducing POC devices to extend TDM as widely as needed in clinical practice. For adoption in patient care, effective and efficient POC devices must be light, affordable, and easy to use. However, not all the technologies for detection and quantification are suitable for this purpose. Liquid chromatography and mass spectroscopy are perfectly suited for the TDM as currently performed,^34^ as they enable multipanel detection at extremely low concentrations with high precision.^35^ However, they remain limited to hospital laboratories because, though cost-effective, their typical sizes and complexity-in-use allow applications to centralized, remote analytical settings only.^36^ Efforts toward miniaturization of the reference methods are warranted. However, magnetic resonance remains a delicate and demanding technology, which might probably limit its potential for large-scale implementation in POC systems.^37,38^ Therefore, alternative detection approaches are warranted for POC devices, which might be used for TDM in hospital wards, day-hospital facilities, and outpatient clinics, albeit at the price of lower accuracy.^39^ According to the FDA, EMA, and ICH bioanalytical method validation guidelines,^40^ acceptable accuracy and precision for TDM measurement methods are traditionally defined as relative error or imprecision not exceeding 15% (20% near the limit of quantification). Such performance requirements justify the assay methods used during the clinical development of drugs. However, more flexible acceptance criteria may still be clinically appropriate for specific POC methods when considering the PK and PD variability of the relevant drug, as has been performed for glucometers.^41^ With this in mind, we can examine multiple options that the vast scientific literature offers for new POC technologies in TDM.

The first option involves electrochemical sensors, as highlighted by the enormous success of glucometers, which are widely used among diabetic patients.^42^ They are generally relatively inexpensive, portable, sensitive enough for many applications in TDM,^43^ and have been suggested for the detection of therapeutic drugs, both for direct and enzyme-mediated detection.

In the first case, electrochemical detection is performed using a bare electrode because the target compound is directly electrochemically active.^44^ Therefore, it is prone to a direct redox reaction at the sensor interface, where electrical charge exchange occurs and is detected by an amperometric measurement. For example, the direct amperometric measurement of anticancer agents (eg, etoposide and methotrexate^44,45^), antipyretics (paracetamol^46^), antidepressants (citalopram^47^), and anesthetics (propofol and midazolam^46,48^) has been successfully demonstrated across the corresponding range of clinical concentrations. This technology has been applied to fabricated aqueous samples and undiluted human serum,^46,48^ demonstrating the applicability of these methods to patient samples. Although the redox reaction itself is not specific, cyclic voltammetry offers a fair degree of specificity for the target analyte by measuring the current peaks occurring at voltage transitions specific to the molecule.

In the second case, the therapeutic compound is not prone to direct redox reaction at the sensor interface. Therefore, a redox enzyme is required to ensure a measurable current from redox reactions involving the therapeutic agent.^49^ In this case, specific enzymes are used, such as cytochrome P450 isoenzymes specifically involved in the metabolism of the drug in question.^49,50^ Several therapeutic drugs have already been detected with this approach, such as psychotropic agents (benzphetamine^51^), anticancer chemotherapies (eg, cyclophosphamide, etoposide, ifosfamide, and ftorafur^52^), kinase inhibitors (lapatinib^53^), antibiotics (ofloxacin^54^), cough suppressants (dextromethorphan^55^), and anti-inflammatory drugs (naproxen and flurbiprofen^55^). Another approach relies on drug-dependent immobilization of glucose-oxidase close to the sensing electrode, achieved by coupling it with an appropriate ligand; beta-lactam antibiotics can be measured this way using a biosensor functionalized with bacterial penicillin-binding protein.^56^

Electrode-bound aptamers functionalized with a redox label (eg, methylene blue) have been used to ensure the selectivity of electrochemical detection, such as for vancomycin,^57^ cocaine,^58^ phenylalanine,^59^ or carbamazepine.^60^ Electrode functionalization extends the range of drugs measurable by electrochemical analysis and enhances analytical specificity, in addition to modifying the shape of the cyclic voltammogram. These electrochemical approaches have several potential applications, such as anticancer,^52^ anti-bacterial,^54^ anti-tuberculosis,^61^ and anti-inflammatory treatments,^55^ as well as anesthesiology.^48^ The list of therapeutic compounds that may be detected with electrochemical biosensors is long.^62^ By exploiting some of these options, it is easy to develop POC devices for fast TDM at the patient's bed. For example, the possibility of realizing an all-in-one device for propofol monitoring, easy to handle during surgery, has been demonstrated.^63^ The proposed device integrates an electrochemical sensor and all the electronic frontend and backend for the autonomous measurement of propofol and the automatic transmission of data to a remote station.^63^ What's more, these sensors also enable frequent, virtually continuous blood monitoring, as shown in the case of propofol^64^ and naproxen.^65^

The second main option of POC technology is offered by optical sensors based on antibodies,^66^ artificial aptamers,^67,68^ or drug receptors.^69^ A well-known example is represented by pregnancy tests accessible at a pharmacy at a negligible cost.^70^ The typical optical sensor for a pregnancy test is based on lateral-flow technology, and it provides positive/negative evaluations only. However, this technology may be pushed to quantitative measurements as well.^71^ Therefore, it is possible to examine the numerous options that the scientific literature offers for POC technologies in TDM using optical detection approaches.^72^ In the case of optical technologies, the taxonomy may focus on the way the optical signal is generated. For example, lamotrigine has been measured using fluorescence,^73^ tobramycin using polarization fluorescence,^74^ levofloxacin using ultraviolet spectrophotometry,^75^ paracetamol using chemiluminescence,^76^ carbamazepine using UV absorption,^77^ imatinib or tobramycin using surface plasmon resonance (SPR),^78,79^ methotrexate or theophylline using bioluminescence,^80,81^ aripiprazole using nanoparticle immunoaggregation,^82^ and monoclonal antibodies using miniaturized enzyme-based immunoassay^83^ or immunofluorescence.^84^ Naproxen has been detected using chemiluminescence^85^ and SPR,^86^ and methotrexate using fluorimetry,^87^ Raman spectroscopy,^88^ and SPR.^89^ The list of therapeutic compounds that may be detected by optical biosensors is very long.^72^ Some of these optical technologies might be implemented in POC devices, as already demonstrated for the measurement of infliximab using fluorescence,^90^ clozapine using immunoassay,^91^ or adalimumab using SPR.^92^ An attractive strategy to implement optical sensing technologies into POC devices is to oversimplify the system, as shown in the case of TDM in oncology.^93^

Either way (electrochemical or optical), the possibility of developing portable, user-friendly, and cost-effective POC devices for TDM has been largely demonstrated.^94^ Conversely, the electrochemical and optical biosensors are not the only technologies to precisely quantify therapeutic drugs using detection approaches that allow for integration on a scale down to POC. There are several options, and the aim of this article is not to provide an exhaustive list of all these options; papers specifically reviewing this field are available in the literature.^62,72^ However, other options should also be mentioned briefly herein to illustrate the use of both well-established technologies already introduced to the market (even for other applications) and new emerging technologies with unprecedented detection capabilities (even though they have been demonstrated for different kinds of chemicals). An approach based on ion-sensitive field-effect transistors (ISFETs) is well established. Indeed, with this technology, several therapeutic compounds have been quantified in human samples, including those not limited to heparin,^95^ naproxen,^96^ and tenofovir.^67^ Moreover, the ISFET technology is well established and largely proposed by the market for various applications such as sequencing,^97^ having demonstrated scale capabilities down to integrating more than hundreds of thousands of sensors in a single platform with a size of 6.4 × 3.9 mm only.^98^ Oppositely, in the category of emerging technologies, the case of memristive biosensors should be mentioned. Memristive biosensing technology was first introduced in the scientific literature approximately 15 years ago,^99^ mainly due to the exploitation of a new physical phenomenon, the memristive effect, first discovered in 2008 in switching materials, such as titanium oxide,^100^ and confirmed in 2010 in silicon nanowires.^101^ It can be roughly defined as a varying resistance in an electrically conductive material that shows a dynamic relationship between current and voltage, including the memory of past charges and currents. In particular, the memristive effect gives rise to a new type of biosensor when the charges carried by biomolecules in physiological conditions interact with the conductivity at the surface of a nanowire.^102^ With this approach, highly sensitive biosensing for measuring prostate-specific antigen (PSA) in human serum has been demonstrated, with an unprecedented limit of detection, down to a few tens of attomoles.^103^ The same approach has been used to develop suitably sensitive biosensors for the TDM of tenofovir^104^ and recently imatinib.^105^ Moreover, previous studies have demonstrated the possibility of integrating such new sensors in multipanel POC^106^ and the application of such POC to clinical samples.^107^

In addition, the sampling technique itself poses a challenge in supporting possible home collection, as the measurement is performed either in a laboratory or with a portable device on the patient's side. For instance, dried blood spots (DBSs), proposed in the 1980s, constitute an attractive technique for patients in TDM.^108,109^ Indeed, DBS allows for reduced sample volume, minimally invasive collection, improved transport logistics, and patient preference.^110^ However, DBS methods that use filter cards have several drawbacks, including contamination, inconsistent sample volume/spot area, and inhomogeneous blood distribution on the card, which can lead to bias in drug quantification.^111^ To overcome these limitations, new techniques have been developed with superior potential for sample integrity, such as volumetric absorptive microsampling (VAMS) tips,^110^ an easy and patient-friendly sampling method that requires only a small volume of blood through a finger prick, allowing patients to perform home sampling.^112^

The scenario presented in this section thus demonstrates that scientific literature, as well as the market in some cases, reports several technological approaches for developing POC solutions that are sufficiently precise and cost-effective for TDM applications. Biosensors with high precision^52^ have been integrated into POC devices^80^ and are capable of quantifying drugs in human and clinical samples.^96,107^

## MODEL-INFORMED PRECISION DOSING: SUITABLE DEVELOPMENTS

**FIGURE 1.**
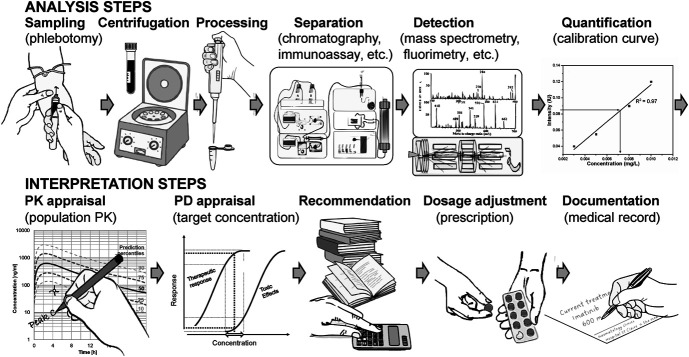
Schematic overview of the elementary stages of a traditional therapeutic drug monitoring process.

Bedside drug measurement through POC technologies promises to shorten the long turnaround time of laboratory analyses causing significant problems in terms of dosage adjustment, especially in clinical centers that do not benefit from specialized on-site facilities.^17,94,113,114^ While POC analyses are approaching clinical use, the quantification of drug exposure as a surrogate for therapeutic response is meaningless without expert pharmacologic interpretation and consequent dosage adjustment recommendation.^17^ Until now, even if expert opinion is available on-site, the traditional TDM workflow faces several limitations for efficient dosage adaptation (Fig. 1). Indeed, a steady state of drug treatment needs to be attained; sampling must be performed at specific, predefined time points to determine peak and/or trough concentrations, and dosage adaptation recommendations must be made manually, often based on the rule of 3 calculations. Traditional TDM cannot utilize the area under the curve (AUC), a PK indicator that is more complex to obtain than the peak or trough concentration. However, it is better correlated with clinical outcomes for several drugs (eg, vancomycin^115^ and rifampicin^116^).

A notable advancement in this field is the emergence of MIPD. This set of approaches uses mathematical and statistical models developed using population methodologies to characterize inter- and intra-individual variability while identifying significant patient-specific covariates,^117,118^ to provide both a priori and a posteriori recommendations for dosage adaptation. Indeed, supplying the MIPD tool with patient characteristics enables it to make a priori predictions of the drug's PK in a specific patient, based on relevant covariates. Such a priori knowledge can guide clinicians to a more appropriate dosage before any TDM sample is taken, provided that the model appropriately fits the target population. Sampling can then be performed at virtually any time owing to the maximum a posteriori Bayesian estimation, which allows for maximum likelihood estimation of the entire concentration curve over time, including the AUC and extrapolations of peak and trough levels, thereby eliminating the practical constraint of time-specific sampling. Dosage adaptation recommendations are then determined by comparing the predicted PK indicator with the corresponding target, enabling the clinician to select 1 option over another by comparing the model predictions with alternative dosage recommendations. This approach, now implemented using several software tools,^119^ fully guides clinicians through the decision-making process, allowing them to focus on selecting the best treatment option for their patients.

Population PK modeling is at the heart of MIPD, and selecting the model suitable for the right patient population is crucial, especially for a priori dosage regimen suggestions,^120^ and includes criteria such as sex, age, body size, ethnicity, comedications, indication for treatment, and comorbidities.^121^ Several published models may compete for a given drug in a defined patient population, leaving clinicians with the challenging task of selecting the most suitable model for the target population. Some MIPD software packages tackle this issue by designing model-selection algorithms to guide clinicians into this nontrivial task, such as InsightRX GEMINI^SM^ (InsightRX, San Francisco, CA) for vancomycin model selection.^122^ An automated continuous learning approach has been proposed to tailor existing models to a different patient population. As more samples become available, the model is updated to better fit the new targeted patients.^123^ This proposed strategy can expedite the lengthy and complex process of developing population PK models tailored to specific patient populations for drugs with a rich and coherent PK literature. Another approach to circumvent the challenging step of model selection for MIPD involves combining multiple models in a model-averaging approach.^124^ Interestingly, in the context of vancomycin and infliximab, the model-averaging method outperforms model selection in terms of a posteriori predictions; yet, this is not suitable for a priori dose recommendations due to the lack of informative samples.^125,126^ In the latter situation, multimodel selection and ensembling approaches have been proposed to select the best evidence supporting adequate a priori dosage individualization: retrospective TDM measurements from a prior population are used to externally evaluate existing drug models, which are then retained and weighted based on their fitting performances to provide an initial recommendation for dosage.^127^ MIPD is expected to integrate with other emerging forms of AI.^128^ These considerations highlight the fact that the “model” part of MIPD is the main driver of the “precision” aspect and thus warrants careful consideration.

The applications of MIPD are not limited to PK-only approaches but may also extend to treatment outcomes by integrating PK-PD models with assessment of efficacy and/or tolerability biomarkers.^129^ Indeed, drug concentration is associated with therapeutic response, but it cannot fully evaluate it. In infectiology, endogenous biomarker measurements, such as interleukins or procalcitonin, can facilitate the monitoring of disease progression, thereby enabling the further refinement of dosages in terms of efficacy.^130^ In the same context, biomarkers for nephrotoxicity, for example, could provide considerable advantages over the standard kidney function evaluation by providing early warnings of organ damage, possibly preventing permanent effects.^130^ Together with drug exposure, biomarkers can provide a more complete view of the treatment efficacy/toxicity in the context of specific diseases, enabling the refinement of MIPD dosage recommendations.

The current state of MIPD, as well as its perspectives, has recently been thoroughly reviewed.^7^ Unsurprisingly, the benefits of MIPD over traditional TDM are difficult to assess, as many studies have been conducted using retrospective data,^120^ while few prospective clinical studies have been reported.^131^ As described in Section 6, various MIPD software tools are available, differing in their underlying calculation methods, costs, quality control, number of drug modules, user interface, software features, and user support. Although generally well-appraised by precision dosing experts, the lack of rigorous studies to assess the clinical and economic benefits of MIPD hinders its widespread implementation in clinical practice.^132^

The future of MIPD is therefore tightly dependent on both clinical research and technological innovation. Prospective studies using MIPD remain warranted, and are feasible due to the availability of population PK models, MIPD software packages, and model selection/averaging approaches.^130^ Moreover, for MIPD to demonstrate its maximum benefits, TDM sampling should be performed in the most efficient way possible for rapid a posteriori intervention, with limited turnaround time, as POC devices seem to promise.^7^ This aspect raises interest in the ultimate outlook of closed-loop dosage adjustment: beyond short-loop TDM, POC technologies for continuous exposure monitoring could enable infusion pumps to adjust automatically, thanks to MIPD-based control, providing real-time, continuous, personalized dose adjustments at the bedside. Promising achievements have been accomplished with antibiotics,^7,133–135^ anticancer agents,^136–138^ and other drugs.^94,139^ Continuous TDM at the infection site might even become conceivable, as indicated by the possibility of following vancomycin concentration in the cerebrospinal fluid.^140^

Therefore, beyond its potential in current TDM practices, MIPD has become a key approach for making TDM accessible at the patient's bedside. Together with POC technologies for drug exposure measurements, closed-loop TDM and continuous, real-time adjusted drug delivery can have a profound impact on clinical practice. As the fields of POC and MIPD have gained traction in research, their joint implementation in marketed devices, following rigorous validation through prospective clinical studies, is an outlook that could ultimately be key to making TDM widely accessible.

## DIGITALIZATION OF THERAPEUTIC DECISIONS: FORTHCOMING EVOLUTION

While traditional MIPD software relies on Bayesian forecasting, machine learning (ML) is attracting attention in the digitalization of therapeutic decision-making, enabling data-driven optimization of treatment regimens and improving patient outcomes.^141^ These technologies have found applications in both precision dosing and POC diagnostic systems. By analyzing patient-specific data, including genetic markers, organ function, and medication history, ML-based models can facilitate precise and dynamic therapeutic interventions. In addition, ML-driven CDSS refine drug dosing recommendations based on real-world evidence and clinical trials, ensuring optimal therapeutic efficacy while minimizing adverse drug reactions. Two key branches of ML applications in therapeutic decision-making can be considered: (1) as a dosage adjustment tool for individualized medicine, and (2) as an integral component of POC devices for real-time data processing.

The emergence of ML has advanced precision dosing, particularly through MIPD, which extends the principles of computational TDM by integrating ML-based pattern recognition with Bayesian inference and dynamically refining dosing recommendations based on observed drug concentrations.^142,143^ Unlike traditional approaches, ML algorithms can continuously learn from patient data by capturing complex nonlinear relationships and improving dosing precision over time. Various ML-based methods, such as the approach of You et al for imatinib,^144^ have demonstrated success in optimizing drug regimens for over a decade. Supervised learning models, such as neural networks and support vector machines (SVMs), have indeed been used to predict optimal drug doses by analyzing large-scale PK/PD datasets. Recently, Li et al^145^ described an ML workflow for dose individualization, detailing critical steps, such as study design, data collection, model training, validation, and clinical implementation. Similarly, reinforcement learning (RL) has shown promise in adaptive dosing strategies, particularly in oncology, where anticancer drug regimens require real-time modifications. In a scoping review, Teplytska et al^146^ highlighted the potential of RL in precision dosing and demonstrated its ability to optimize drug administration schedules based on continuous patient monitoring. Moreover, ML-based predictive analytics has been successfully applied to TDM. Yoon et al^147^ recently developed and validated an ML model for the precise dosing of tacrolimus in liver transplant recipients, utilizing long short-term memory (LSTM) and gradient-boosted regression tree (GBRT) models trained on time-series dose-concentration data and clinical covariates, demonstrating improved predictive performance over conventional dosing strategies.

Beyond dosage adjustment, ML also transforms the capabilities of POC devices by automating data acquisition, signal processing, and clinical decision-making. Portable biosensors, diagnostic assays, and wearable health monitors generate vast amounts of physiological and biochemical data requiring rapid analysis. ML-driven POC devices enhance the diagnostic accuracy, automate anomaly detection, and enable continuous patient monitoring. AI-enhanced systems leverage automated image and signal processing techniques to analyze biosensor data, medical imaging, and laboratory test results. For instance, a POC system integrating contrast-enhanced microholography and convolutional neural networks (CNNs) has demonstrated high accuracy in lymphoma classification by analyzing fine-needle aspirates, reducing reliance on specialized pathology resources, and making high-precision diagnostics accessible, even in resource-limited settings.^148^ Moreover, multifrequency impedance cytometry combined with supervised ML models has improved drug efficacy evaluations, particularly in targeted cancer therapy. By analyzing cellular dielectric properties and employing SVMs, POC devices can classify tumor cell viability without staining or labeling, enhancing accuracy and workflow efficiency.^149^ In addition, ML has significantly advanced electrochemical sensor applications in POC devices, enabling the rapid and accurate identification and quantification of multiple drugs from cyclic voltammogram redox peak curves.^150,151^

**FIGURE 2.**
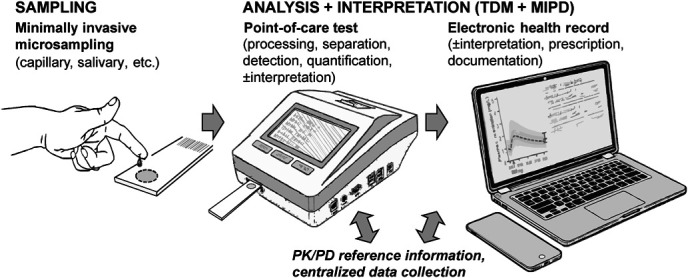
Combined stages of integrated therapeutic drug monitoring relying on a point-of-care system.

Therefore, ML-driven MIPD and POC technologies are likely to shape the future of individualized treatment (Fig. 2). By integrating drug exposure monitoring with adaptive dosing algorithms, ML facilitates closed-loop TDM, enabling dynamic and continuous drug adjustments at a patient's bedside.^142^ As advancements in MIPD and POC technologies accelerate, their combined implementation in clinical practice has the potential to redefine precision medicine, making TDM accessible, scalable, and widely applicable in diverse healthcare settings.

In terms of data management, the data must be transmitted from the POC sample value to the MIPD dosage adaptation. This can be accomplished directly from the POC to the MIPD software if both systems are interconnected, or through an institution's EHR, allowing the MIPD software to gather all the required data. While direct connection is a potential option, full integration is the preferred choice for traditional institutions where gathering data from POCs is essential.^152–154^ POC interoperability reduces the risk of transcription errors, improves reliability, and facilitates integration with EHRs.^155,156^ The POCT1-A2 ISO standard^157^ can efficiently manage communication and data structures.^158^

Connectivity is also crucial from the EHR to the MIPD software. Standards, notably HL7 FHIR and OpenEHR,^159^ aim to enable data exchange between different systems. However, they suffer from genericity. Based on HL7, IHE is designed to facilitate real data interchange for particular clinical and operational purposes domains.^160^ However, TDM is not yet part of the supported domains. Another difficulty lies in integrating a significant number of EHRs, each of which offers a different method of connection. When developing POCs or MIPD software, 1 key issue is determining whether to develop an in-house solution or utilize existing software solutions. A hybrid approach aiming at standardizing the system inputs/outputs and allowing an intermediate gateway, such as Mirth Connect, to link with the EHR represents a good solution.^161^

However, the quality of the data present in the EHR is crucial.^162,163^ Typically, institutions should impose numeric fields where a value is expected (dose value, duration, and measurement) and only authorize valid units in the corresponding fields. Indeed, systems that are too lax and only store free fields may simplify data entry for staff but significantly hinder automated data processing and retrieval for monitoring purposes. Ultimately, human factors are always important. For instance, if a nurse does not accurately record the time of sampling, the entire TDM process can be compromised.^164^ In this context, future POC systems should store the current time when processing a sample, as it is likely to be close to the time of blood sampling and would help prevent issues with incorrect sampling time inputs.

## CONCLUSION, GAPS, AND PERSPECTIVES

TDM has been restricted to a few classical drugs by measuring their concentrations in analytical laboratories. Emerging evidence suggests that TDM can expand owing to POC devices and related digital technologies. Several promising approaches have been described for applying POC in TDM.

Portable sensors offer a unique opportunity to significantly shorten the feedback loop from drug prescriptions to dosage adjustments. Although this path involves biological sampling, POC analysis, MIPD-supported interpretation, and advice on dosage adjustment, all the required technologies can now be integrated into fully connected POC devices. This opens up the perspective of developing TDM as an integrated component of a highly advanced digital healthcare system for patient management. Key elements of advanced digital health care are mathematical models, which are developed using population approaches to characterize inter- and intra-individual PK variability and are required to identify patient-specific covariates to provide a priori and a posteriori recommendations for dosage adjustment. A key to success is the ability to manage this complexity using digital technology to dynamically refine the dosing recommendations.

Multiple challenges remain to be addressed to bridge the gap between current knowledge and technology and the future potential they hold for TDM at the point of care. These include limited research resources, pharmaceutical companies' relative lack of interest, regulatory issues, difficulties in adoption by healthcare professionals, and various technological obstacles that complicate the large-scale development of POC systems for TDM. However, enumerating these hurdles in detail falls outside the scope of this narrative review. Nevertheless, technical and medical progress is expected to continue, which will significantly benefit precise drug dosing. This may involve the use of graphical interfaces, seamless connectivity with EHR, and ML approaches to further advance the principles of computation in TDM, for example, by integrating AI-based pattern recognition with Bayesian inference.

Opportunities currently exist to extend TDM to a broad range of drugs and provide precise dosing to numerous healthcare professionals and their patients.

## References

[1] Raman K Kumar R Musante CJ . Integrating model-informed drug development with AI: a synergistic approach to accelerating pharmaceutical innovation. Clin Transl Sci. 2025;18:e70124. 39797502 10.1111/cts.70124 PMC11724156

[2] Xiong Y Samtani MN Ouellet D. Applications of pharmacometrics in drug development. Adv Drug Deliv Rev. 2025;217:115503. 39701388 10.1016/j.addr.2024.115503

[3] Darwich AS Ogungbenro K Vinks AA . Why has model-informed precision dosing not yet become common clinical reality? Lessons from the past and a roadmap for the future. Clin Pharmacol Ther. 2017;101:646–656. 28182269 10.1002/cpt.659

[4] Peck RW. Precision dosing: an industry perspective. Clin Pharmacol Ther. 2021;109:47–50. 33107023 10.1002/cpt.2064 PMC7820949

[5] Tyson RJ Park CC Powell JR . Precision dosing priority criteria: drug, disease, and patient population variables. Front Pharmacol. 2020;11:420. 32390828 10.3389/fphar.2020.00420 PMC7188913

[6] Gonzalez D Rao GG Bailey SC . Precision dosing: public health need, proposed framework, and anticipated impact. Clin Transl Sci. 2017;10:443–454. 28875519 10.1111/cts.12490 PMC5698804

[7] Minichmayr IK Dreesen E Centanni M . Model-informed precision dosing: state of the art and future perspectives. Adv Drug Deliv Rev. 2024;215:115421. 39159868 10.1016/j.addr.2024.115421

[8] Wang L Maxfield K Guinn D . A systematic assessment of US food and drug administration dosing recommendations for drug development programs amenable to response-guided titration. Clin Pharmacol Ther. 2021;109:123–130. 33022770 10.1002/cpt.2068 PMC7902398

[9] Collins FS Varmus H. A new initiative on precision medicine. N Engl J Med. 2015;372:793–795. 25635347 10.1056/NEJMp1500523 PMC5101938

[10] Jang SH Yan Z Lazor JA. Therapeutic drug monitoring: a patient management tool for precision medicine. Clin Pharmacol Ther. 2016;99:148–150. 26565378 10.1002/cpt.298

[11] Buclin T Thoma Y Widmer N . The steps to therapeutic drug monitoring: a structured approach illustrated with imatinib. Front Pharmacol. 2020;11:177. 32194413 10.3389/fphar.2020.00177 PMC7062864

[12] Buclin T Gotta V Fuchs A . Monitoring drug therapy. Br J Clin Pharmacol. 2012;73:917–923. 22360377 10.1111/j.1365-2125.2012.04237.x PMC3391519

[13] Buclin T Widmer N Biollaz J . Who is in charge of assessing therapeutic drug monitoring? The case of imatinib. Lancet Oncol. 2011;12:9–11. 21111679 10.1016/S1470-2045(10)70258-8

[14] Oles KS. Therapeutic drug monitoring analysis systems for the physician office laboratory: a review of the literature. DICP (Ann Pharmacother). 1990;24:1070–1077. 10.1177/106002809002401111 2275232

[15] Stradolini F Lavalle E De Micheli G . Paradigm-shifting players for IoT: smart-Watches for intensive care monitoring. In: Perego P Andreoni G Rizzo G eds Wireless Mobile Communication and Healthcare: Proceedings of the 6^th^ International Conference, MobiHealth 2016 (Milan, Italy, November 14-16, 2016). Heidelberg: Springer International Publishing; 2017:71–78.

[16] Sanavio B Krol S. On the slow diffusion of point-of-care systems in therapeutic drug monitoring. Front Bioeng Biotechnol. 2015;3:20. 25767794 10.3389/fbioe.2015.00020 PMC4341557

[17] Ates HC Roberts JA Lipman J . On-site therapeutic drug monitoring. Trends Biotechnol. 2020;38:1262–1277. 33058758 10.1016/j.tibtech.2020.03.001

[18] Koch BCP Muller AE Hunfeld NGM . Therapeutic drug monitoring of antibiotics in critically ill patients: current practice and future perspectives with a focus on clinical outcome. Ther Drug Monit. 2022;44:11–18. 34772892 10.1097/FTD.0000000000000942

[19] Märtson AG Burch G Ghimire S . Therapeutic drug monitoring in patients with tuberculosis and concurrent medical problems. Expert Opin Drug Metab Toxicol. 2021;17:23–39. 33040625 10.1080/17425255.2021.1836158

[20] Cattaneo D Gervasoni C. Therapeutic drug monitoring of antiretroviral therapy: current progresses and future directions. Expert Rev Clin Pharmacol. 2024;17:579–587. 38829318 10.1080/17512433.2024.2363847

[21] Gómez-López A Antifungal therapeutic drug monitoring: focus on drugs without a clear recommendation. Clin Microbiol Infect. 2020;26:1481–1487. 32535150 10.1016/j.cmi.2020.05.037

[22] Johannessen Landmark C Johannessen SI Patsalos PN. Therapeutic drug monitoring of antiepileptic drugs: current status and future prospects. Expert Opin Drug Metab Toxicol. 2020;16:227–238. 32054370 10.1080/17425255.2020.1724956

[23] Paci A Veal G Bardin C . Review of therapeutic drug monitoring of anticancer drugs: part 1 – cytotoxics. Eur J Cancer. 2014;50:2010–2019. 24889915 10.1016/j.ejca.2014.04.014

[24] Mueller-Schoell A Groenland SL Scherf-Clavel O . Therapeutic drug monitoring of oral targeted antineoplastic drugs. Eur J Clin Pharmacol. 2021;77:441–464. 33165648 10.1007/s00228-020-03014-8 PMC7935845

[25] Schoretsanitis G Kane JM Correll CU . Blood levels to optimize antipsychotic treatment in clinical practice: a joint consensus statement of the American Society of Clinical Psychopharmacology and the Therapeutic Drug Monitoring Task Force of the Arbeitsgemeinschaft für Neuropsychopharmakologie und Pharmakopsychiatrie. J Clin Psychiatry. 2020;81:19cs13169. 10.4088/JCP.19cs13169 32433836

[26] Irving PM Gecse KB. Optimizing therapies using therapeutic drug monitoring: current strategies and future perspectives. Gastroenterology. 2022;162:1512–1524. 35167865 10.1053/j.gastro.2022.02.014

[27] Galfo V Tiseo G Riccardi N . Therapeutic drug monitoring of antibiotics for methicillin-resistant Staphylococcus aureus infections: an updated narrative review for clinicians. Clin Microbiol Infect. 2025;31;194–200. 39209264 10.1016/j.cmi.2024.08.021

[28] Choong E Sauty A Koutsokera A . Therapeutic drug monitoring of ivacaftor, lumacaftor, tezacaftor, and elexacaftor in cystic fibrosis: where are we now? Pharmaceutics. 2022;14:1674. 36015300 10.3390/pharmaceutics14081674 PMC9412421

[29] García-Calderón CB Sierro-Martínez B García-Guerrero E . Monitoring of kinetics and exhaustion markers of circulating CAR-T cells as early predictive factors in patients with B-cell malignancies. Front Immunol. 2023;14:1152498. 37122702 10.3389/fimmu.2023.1152498 PMC10140355

[30] Briki M André P Thoma Y . Precision oncology by point-of-care therapeutic drug monitoring and dosage adjustment of conventional cytotoxic chemotherapies: a perspective. Pharmaceutics. 2023;15:1283. 37111768 10.3390/pharmaceutics15041283 PMC10147065

[31] Horwitz RI Hayes-Conroy A Caricchio R . From evidence based medicine to medicine based evidence. Am J Med. 2017;130:1246–1250. 28711551 10.1016/j.amjmed.2017.06.012

[32] Gotta V Widmer N Montemurro M . Therapeutic drug monitoring of imatinib: bayesian and alternative methods to predict trough levels. Clin Pharmacokinet. 2012;51:187–201. 22339450 10.2165/11596990-000000000-00000

[33] Cafaro A Conti M Pigliasco F . Biological fluid microsampling for therapeutic drug monitoring: a narrative review. Biomedicines. 2023;11:1962. 37509602 10.3390/biomedicines11071962 PMC10377272

[34] Briki M Murisier A Guidi M . Liquid chromatography coupled to tandem mass spectrometry (LC-MS/MS) methods for the therapeutic drug monitoring of cytotoxic anticancer drugs: an update. J Chromatogr B, Anal Tech Biomed Life Sci. 2024;1236:124039. 10.1016/j.jchromb.2024.124039 38490042

[35] Qi Y Liu G. A UPLC-MS/MS method for simultaneous determination of eight special-grade antimicrobials in human plasma and application in TDM. J Pharm Biomed Anal. 2022;220:114964. 36084471 10.1016/j.jpba.2022.114964

[36] Tuzimski T Petruczynik A. Review of chromatographic methods coupled with modern detection techniques applied in the therapeutic drugs monitoring (TDM). Molecules. 2020;25:4026. 32899296 10.3390/molecules25174026 PMC7504794

[37] Pandey S Hu Y Bushman LR . Miniature mass spectrometer-based point-of-care assay for cabotegravir and rilpivirine in whole blood. Anal Bioanal Chem. 2022;414:3387–3395. 35169905 10.1007/s00216-022-03954-3 PMC9018536

[38] Chen J Li Y Chen J . Miniature mass spectrometer-based point-of-care assay for quantification of metformin and sitagliptin in human blood and urine. Anal Bioanal Chem. 2024;416:3305–3312. 38642098 10.1007/s00216-024-05281-1

[39] Taddeo A Prim D Bojescu ED . Point-of-care therapeutic drug monitoring for precision dosing of immunosuppressive drugs. J Appl Lab Med. 2020;5:738–761. 32533157 10.1093/jalm/jfaa067

[40] ICH, Guideline M10 - bioanalytical method validation and Study sample analysis. 2022. Available at: https://database.ich.org/sites/default/files/M10_Guideline_Step4_2022_0524.pdf. Accessed June 2025.

[41] Clarke WL. The original Clarke Error Grid Analysis (EGA). Diabetes Technol Ther. 2005;7:776–779. 16241881 10.1089/dia.2005.7.776

[42] Clarke SF Foster JR. A history of blood glucose meters and their role in self-monitoring of diabetes mellitus. Br J Biomed Sci. 2012;69:83–93. 22872934

[43] Pollard TD Ong JJ Goyanes A . Electrochemical biosensors: a nexus for precision medicine. Drug Discov Today. 2021;26:69–79. 33137482 10.1016/j.drudis.2020.10.021

[44] Rodino F Bartoli M Carrara S. Simultaneous and selective detection of etoposide and methotrexate with single electrochemical sensors for therapeutic drug monitoring. IEEE Sensors Lett. 2023;7:1–4.

[45] Liu H Liu YW Yang RY . Therapeutic drug monitoring of methotrexate by disposable SPCE biosensor for personalized medicine. Anal Chim Acta. 2025;1335:343473. 39643323 10.1016/j.aca.2024.343473

[46] Aliakbarinodehi N Stradolini F Nakhjavani SA . Performance of carbon nano-scale allotropes in detecting midazolam and paracetamol in undiluted human serum. IEEE Sensors J. 2018;18;5073–5081.

[47] Chen R Amirghasemi F Ma H . Toward personalized treatment of depression: an affordable citalopram test based on a solid-contact potentiometric electrode for at-home monitoring of the antidepressant dosage. ACS Sens. 2023;8:3943–3951. 37734027 10.1021/acssensors.3c01545 PMC11446579

[48] Stradolini F Kilic T Taurino I . Cleaning strategy for carbon-based electrodes: Long-term propofol monitoring in human serum. Sensors Actuators B: Chem. 2018;269:304–313.

[49] Yarman A Wollenberger U Scheller FW. Sensors based on cytochrome P450 and CYP mimicking systems. Electrochimica Acta. 2013;110:63–72.

[50] Shumyantseva VV Bulko TV Kuzikov AV . All-electrochemical nanocomposite two-electrode setup for quantification of drugs and study of their electrocatalytical conversion by cytochromes P450. Electrochimica Acta. 2020;336:135579.

[51] Carrara S Cavallini A Garg A . Dynamical spot queries to improve specificity in P450s based multi-drugs monitoring. 2009 ICME International Conference on Complex Medical Engineering (Tampe, Arizona, April 9-11, 2009). IEEE; 2009:548–553.

[52] Baj-Rossi C De Micheli G Carrara S. Electrochemical detection of anti-breast-cancer agents in human serum by cytochrome P450-coated carbon nanotubes. Sensors. 2012;12:6520–6537. 22778656 10.3390/s120506520 PMC3386755

[53] January JL Tshobeni ZZ Ngema NPP . Novel cytochrome P450-3A4 enzymatic nanobiosensor for lapatinib (a breast cancer drug) developed on a poly(anilino-co-4-aminobenzoic acid-green-synthesised indium nanoparticle) platform. Biosensors. 2023;13:897. 37754131 10.3390/bios13090897 PMC10527071

[54] Kelani KM Fayez YM Gad AG. et al. Design of point-of-care electrochemical sensor for therapeutic drug monitoring of ofloxacin in biological fluids. J Anal Sci Technol. 2024;15:41.

[55] Carrara S Cavallini A Erokhin V . Multi-panel drugs detection in human serum for personalized therapy. Biosens Bioelectron. 2011;26:3914–3919. 21497079 10.1016/j.bios.2011.03.009

[56] Ates HC Mohsenin H Wenzel C . Biosensor-enabled multiplexed on-site therapeutic drug monitoring of antibiotics. Adv Mater. 2022;34:e2104555. 34545651 10.1002/adma.202104555 PMC11468941

[57] Liu Y Mack JO Shojaee M . Analytical validation of aptamer-based serum vancomycin monitoring relative to automated immunoassays. ACS Sens. 2024;9:228–235. 38110361 10.1021/acssensors.3c01868 PMC10826698

[58] Alkhamis O Canoura J Wu Y . High-affinity aptamers for in vitro and in vivo cocaine sensing. J Am Chem Soc. 2024;146:3230–3240. 38277259 10.1021/jacs.3c11350 PMC11849797

[59] Verrinder E Gerson J Leung K . Dual-frequency, ratiometric approaches to EAB sensor interrogation support the calibration-free measurement of specific molecules in vivo. ACS Sens. 2024;9:3205–3211. 38775190 10.1021/acssensors.4c00516 PMC11821477

[60] Chung S Singh NK Gribkoff VK . Electrochemical carbamazepine aptasensor for therapeutic drug monitoring at the point of care. ACS Omega. 2022;7:39097–39106. 36340178 10.1021/acsomega.2c04865 PMC9631757

[61] Franke C Ajayi RF Uhuo O . Metallodendrimer‐sensitised cytochrome P450 3A4 electrochemical biosensor for TB drugs. Electroanalysis. 2020;32:3075–3085.

[62] Teymourian H Parrilla M Sempionatto JR . Wearable electrochemical sensors for the monitoring and screening of drugs. ACS Sensors. 2020;5:2679–2700. 32822166 10.1021/acssensors.0c01318

[63] Aiassa S Ros PM Hanitra MIN . Smart portable pen for continuous monitoring of anaesthetics in human serum with machine learning. IEEE Trans Biomed Circuits Syst. 2021;15:294–302. 33739925 10.1109/TBCAS.2021.3067388

[64] Stradolini F Tuoheti A Kilic T . An IoT solution for online monitoring of anesthetics in human serum based on an integrated fluidic bioelectronic system. IEEE Trans Biomed Circuits Syst. 2018;12:1056–1064. 30072339 10.1109/TBCAS.2018.2855048

[65] Baj-Rossi C Rezzonico Jost T Cavallini A Continuous monitoring of naproxen by a cytochrome P450-based electrochemical sensor. Biosens Bioelectron. 2014;53:283–287. 24144559 10.1016/j.bios.2013.09.058

[66] Sharma S Byrne H O'Kennedy RJ. Antibodies and antibody-derived analytical biosensors. Essays Biochem. 2016;60:9–18. 27365031 10.1042/EBC20150002 PMC4986469

[67] Aliakbarinodehi N Jolly P Bhalla N . Aptamer-based field-effect biosensor for tenofovir detection. Sci Rep. 2017;7:44409. 28294122 10.1038/srep44409 PMC5353720

[68] Garzón V Pinacho DG Bustos RH . Optical biosensors for therapeutic drug monitoring. Biosensors. 2019;9:132. 31718050 10.3390/bios9040132 PMC6955905

[69] Miller RM Sescil J Sarcinella MC . Accessible and generalizable in vitro luminescence assay for detecting GPCR activation. ACS Meas Sci Au. 2023;3:337–343. 37868356 10.1021/acsmeasuresciau.3c00021 PMC10588934

[70] Morris N Ehrenreich K Gurazada T . Feasibility and acceptability of at-home routine pregnancy testing in the United States: a pilot study. Women's Health Issues. 2023;33:258–265. 36822914 10.1016/j.whi.2023.01.002

[71] Mak WC Beni V Turner AP. Lateral-flow technology: from visual to instrumental. Trac Trends Anal Chem. 2016;79:297–305.

[72] Ong JJ Pollard TD Goyanes A . Optical biosensors – illuminating the path to personalized drug dosing. Biosens Bioelectron. 2021;188:113331. 34038838 10.1016/j.bios.2021.113331

[73] Jouyban A Samadi A Khoubnasabjafari M. A new “turn-on” fluorescent sensor based on gold quantum dots and silver nanoparticles for lamotrigine detection in plasma. Talanta. 2017;172:126–132. 28602284 10.1016/j.talanta.2017.05.018

[74] Bojescu ED Prim D Pfeifer ME . Fluorescence-polarization immunoassays within glass fiber micro-chambers enable tobramycin quantification in whole blood for therapeutic drug monitoring at the point of care. Anal Chim Acta. 2022;1225:340240. 36038239 10.1016/j.aca.2022.340240

[75] Mohamed S Mvungi HC Sariko M . Levofloxacin pharmacokinetics in saliva as measured by a mobile microvolume UV spectrophotometer among people treated for rifampicin-resistant TB in Tanzania. J Antimicrob Chemother. 2021;76:1547–1552. 33675664 10.1093/jac/dkab057 PMC8120342

[76] Emdadi S Sorouraddin MH Denanny L. Enhanced chemiluminescence determination of paracetamol. Analyst. 2021;146:1326–1333. 33367305 10.1039/d0an01557a

[77] Ramos II Carl P Schneider RJ . Automated lab-on-valve sequential injection ELISA for determination of carbamazepine. Anal Chim Acta. 2019;1076:91–99. 31203968 10.1016/j.aca.2019.05.017

[78] Fornasaro S Bonifacio A Marangon E . Label-free quantification of anticancer drug imatinib in human plasma with surface enhanced Raman spectroscopy. Anal Chem. 2018;90:12670–12677. 30350602 10.1021/acs.analchem.8b02901

[79] Tenaglia E Ferretti A Decosterd LA . Comparison against current standards of a DNA aptamer for the label-free quantification of tobramycin in human sera employed for therapeutic drug monitoring. J Pharm Biomed Anal. 2018;159:341–347. 30025299 10.1016/j.jpba.2018.06.061

[80] Griss R Schena A Reymond L . Bioluminescent sensor proteins for point-of-care therapeutic drug monitoring. Nat Chem Biol. 2014;10:598–603. 24907901 10.1038/nchembio.1554

[81] Xue L Yu Q Griss R . Bioluminescent antibodies for point-of-care diagnostics. Angew Chem Int Ed Engl. 2017;56:7112–7116. 28510347 10.1002/anie.201702403 PMC5488172

[82] Atkins M Taylor D Catalan A . Point of care assay for blood aripiprazole concentrations: development, validation and utility. Br J Psychiatry. 2023;223:389–393. 37254587 10.1192/bjp.2023.58

[83] Campbell E Adamson H Luxton T . Therapeutic drug monitoring of immunotherapies with novel Affimer-NanoBiT sensor construct. Sens Diagn. 2024;3:104–111. 38249540 10.1039/d3sd00126a PMC10795742

[84] Berwanger JD Tan HY Jokhadze G . Determination of the serum concentrations of the monoclonal antibodies bevacizumab, rituximab, and panitumumab using porous membranes containing immobilized peptide mimotopes. Anal Chem. 2021;93:7562–7570. 33999602 10.1021/acs.analchem.0c04903

[85] Kamruzzaman M Alam AM Kim KM . Silver nanoparticle-enhanced chemiluminescence method for determining naproxen based on europium(III)-sensitized Ce(IV)-Na2S2O4 reaction. J Fluoresc. 2012;22:883–890. 22215565 10.1007/s10895-011-1026-9

[86] Khodaveisi J Shabani AMH Dadfarnia S . A novel sensor for determination of naproxen based on change in localized surface plasmon peak of functionalized gold nanoparticles. Spectrochim Acta A Mol Biomol Spectrosc. 2017;179:11–16. 28213140 10.1016/j.saa.2017.02.008

[87] Ensafi AA Nasr-Esfahani P Rezaei B. Simultaneous detection of folic acid and methotrexate by an optical sensor based on molecularly imprinted polymers on dual-color CdTe quantum dots. Anal Chim Acta. 2017;996:64–73. 29137709 10.1016/j.aca.2017.10.011

[88] Fornasaro S Marta SD Rabusin M . Toward SERS-based point-of-care approaches for therapeutic drug monitoring: the case of methotrexate. Faraday Discuss. 2016;187:485–499. 27055173 10.1039/c5fd00173k

[89] Zhao SS Bichelberger MA Colin DY . Monitoring methotrexate in clinical samples from cancer patients during chemotherapy with a LSPR-based competitive sensor. Analyst. 2012;137:4742–4750. 22943049 10.1039/c2an35839e

[90] Kim ES Chon H Kwon Y . Fluorescence-based lateral flow immunoassay for quantification of infliximab: analytical and clinical performance evaluation. Ther Drug Monit. 2024;46:460–467. 38287890 10.1097/FTD.0000000000001176 PMC11232936

[91] Boland X Dratcu L. First Use of Clozapine point of care testing in acute inpatient psychiatry: preliminary report. J Psychiatr Pract. 2022;28:62–66. 34989347 10.1097/PRA.0000000000000608

[92] Qu JH Ordutowski H Van Tricht C . Point-of-care therapeutic drug monitoring of adalimumab by integrating a FO-SPR biosensor in a self-powered microfluidic cartridge. Biosens Bioelectron. 2022;206:114125. 35255315 10.1016/j.bios.2022.114125

[93] Grosso P Carrara S Stagni C . Cancer marker detection in human serum with a point-of-care low-cost system. Sensors Actuators B Chem. 2010;147:475–480.

[94] Liang WS Beaulieu-Jones B Smalley S . Emerging therapeutic drug monitoring technologies: considerations and opportunities in precision medicine. Front Pharmacol. 2024;15:1348112. 38545548 10.3389/fphar.2024.1348112 PMC10965556

[95] Van Kerkhof JC Bergveld P Schasfoort RB. The ISFET based heparin sensor with a monolayer of protamine as affinity ligand. Biosens Bioelectron. 1995;10:269–282. 7755959 10.1016/0956-5663(95)96846-q

[96] Nemati SS Salemi-Seresht M Abdi Y . Highly sensitive and label-free detection of naproxen using mixed metal oxide-based field effect transistor as a biosensor for in-vitro analysis of urine. Mater Sci Semiconductor Process. 2024;179:108487.

[97] Ma D Rodriguez-Manzano J De Mateo Lopez S . Adapting ISFETs for epigenetics: an overview. IEEE Trans Biomed Circuits Syst. 2018;12:1186–1201. 30010588 10.1109/TBCAS.2018.2838153

[98] Hu K Arcadia CE Rosenstein JK. A large-scale multimodal CMOS biosensor array with 131,072 pixels and code-division multiplexed readout. IEEE Solid-state Circuits Lett. 2021;4:48–51.

[99] Carrara S The birth of a new field: memristive sensors. A review. IEEE Sensors J. 2021;21:12370–12378.

[100] Strukov DB Snider GS Stewart DR . The missing memristor found. Nature. 2008;453:80–83. 18451858 10.1038/nature06932

[101] Sacchetto D Ben-Jamaa MH Carrara S . Memristive devices fabricated with silicon nanowire Schottky barrier transistors. In: 2010 IEEE International Symposium on Circuits and Systems (ISCAS) (Paris, France, May 30-June 2, 2010). IEEE. 2010:9–12.

[102] Puppo F Traversa FL Ventra MD . Surface trap mediated electronic transport in biofunctionalized silicon nanowires. Nanotechnology. 2016;27:345503. 27418560 10.1088/0957-4484/27/34/345503

[103] Tzouvadaki I Jolly P Lu X . Label-free ultrasensitive memristive aptasensor. Nano Lett. 2016;16:4472–4476. 27341189 10.1021/acs.nanolett.6b01648

[104] Tzouvadaki I Aliakbarinodehi N De Micheli G . The memristive effect as a novelty in drug monitoring. Nanoscale. 2017;9:9676–9684. 28675222 10.1039/c7nr01297g

[105] Chen J Alberi L Pétermann Y . Imatinib detection by memristive biosensors for therapeutic drug monitoring, Biosens Bioelectron X. 2025;24:100620.

[106] Tzouvadaki I Tuoheti A De Micheli G . Portable memristive biosensing system as effective point-of-care device for cancer diagnostics. In: *2018 IEEE International Symposium on Circuits and Systems (ISCAS) (Florence, Italy, May 27-30, 2018)*. IEEE. 2018:1–5.

[107] Tuoheti A Pabois A Puppo F . Immune attack against breast cancer revealed in patient tumor sections by memristive nanowire sensors. Br J Cancer Res. 2020;3:341–348.

[108] Spooner N Anderson M Wickremsinhe ER. Patient-centric sampling special focus issue. Bioanalysis. 2020;12:867–868. 32772899 10.4155/bio-2020-0176

[109] Lampe D Scholz D Prümke HJ . Capillary blood, dried on filter paper, as sample for monitoring cyclosporin A concentrations. Clin Chem. 1987;33:1643–1644. 3304715

[110] Leino AD Takyi-Williams J Pai MP. Volumetric absorptive microsampling to enhance the therapeutic drug monitoring of tacrolimus and mycophenolic acid: a systematic review and critical assessment. Ther Drug Monit. 2023;45:463–478. 36728554 10.1097/FTD.0000000000001066

[111] De Kesel PM Sadones N Capiau S . Hemato-critical issues in quantitative analysis of dried blood spots: challenges and solutions. Bioanalysis. 2013;5:2023–2041. 23937137 10.4155/bio.13.156

[112] Verougstraete N Stove CP. Volumetric absorptive microsampling as a suitable tool to monitor tyrosine kinase inhibitors. J Pharm Biomed Anal. 2022;207:114418. 34655987 10.1016/j.jpba.2021.114418

[113] Kim HY et al. Therapeutic drug monitoring of anti-infective drugs: implementation strategies for 3 different scenarios. Ther Drug Monit. 2022;44:3–10. 34686647 10.1097/FTD.0000000000000936 PMC8755585

[114] Leung D Ensom MHH Carr R. Survey of therapeutic drug monitoring practices in pediatric health care programs across Canada. Can J Hosp Pharm. 2019;72:126–132. 31036973 PMC6476573

[115] Rybak MJ Le J Lodise TP . Therapeutic monitoring of vancomycin for serious methicillin-resistant Staphylococcus aureus infections: a revised consensus guideline and review by the American Society of Health-System Pharmacists, the Infectious Diseases Society of America, the Pediatric Infectious Diseases Society, and the Society of Infectious Diseases Pharmacists. Am J Health Syst Pharm. 2020;77:835–864. 32191793 10.1093/ajhp/zxaa036

[116] Alffenaar JWC Stocker SL Forsman LD . Clinical standards for the dosing and management of TB drugs. Int J Tuberc Lung Dis. 2022;26:483–499. 35650702 10.5588/ijtld.22.0188 PMC9165737

[117] Guidi M Csajka C Buclin T. Parametric approaches in population pharmacokinetics. J Clin Pharmacol. 2022;62:125–141. 33103774 10.1002/jcph.1633

[118] Goutelle S Woillard JB Neely M . Nonparametric methods in population pharmacokinetics. J Clin Pharmacol. 2022;62:142–157. 33103785 10.1002/jcph.1650

[119] Del Valle-Moreno P Suarez-Casillas P Mejías-Trueba M . Model-Informed precision dosing software tools for dosage regimen individualization: a scoping review. Pharmaceutics. 2023;15:1859. 37514045 10.3390/pharmaceutics15071859 PMC10386689

[120] Haefliger D Mina L Guidi M . Individualization of piperacillin dosage based on therapeutic drug monitoring with or without model-informed precision dosing: a scenario analysis. J Antimicrob Chemother. 2025;80:840–847. 39821648 10.1093/jac/dkaf007 PMC12086683

[121] Taylor ZL Poweleit EA Paice K . Tutorial on model selection and validation of model input into precision dosing software for model-informed precision dosing. CPT Pharmacometrics Syst Pharmacol. 2023;12:1827–1845. 37771190 10.1002/psp4.13056 PMC10725261

[122] InsightRX Communications. InsightRX announces the release of InsightRX GEMINI model-selection algorithm. Available at: https://blog.insight-rx.com/press-releases/insightrx-gemini-model-selection-algorithm-release Published August 27, 2024. Accessed April 14, 2025.

[123] Hughes JH Tong DMH Lucas SS . Continuous learning in model-informed precision dosing: a case study in pediatric dosing of vancomycin. Clin Pharmacol Ther. 2021;109:233–242. 33068298 10.1002/cpt.2088 PMC7839485

[124] Petit-Jean E Buclin T Guidi M . Erlotinib: another candidate for the therapeutic drug monitoring of targeted therapy of cancer? A pharmacokinetic and pharmacodynamic systematic review of literature. Ther Drug Monit. 2015;37:2–21. 24831652 10.1097/FTD.0000000000000097

[125] Uster DW Stocker SL Carland JE . A model averaging/selection approach improves the predictive performance of model-informed precision dosing: Vancomycin as a case study. Clin Pharmacol Ther. 2021;109:175–183. 32996120 10.1002/cpt.2065

[126] Kantasiripitak W Outtier A Wicha SG . Multi-model averaging improves the performance of model-guided infliximab dosing in patients with inflammatory bowel diseases. CPT Pharmacometrics Syst Pharmacol. 2022;11:1045–1059. 35706358 10.1002/psp4.12813 PMC9381887

[127] Agema BC Kocher T Öztürk AB . Selecting the best pharmacokinetic models for a priori model-informed precision dosing with model Ensembling. Clin Pharmacokinet. 2024;63:1449–1461. 39331236 10.1007/s40262-024-01425-9 PMC11522197

[128] Varela-Rey I Bandín-Vilar E Toja-Camba FJ . Artificial intelligence and machine learning applications to pharmacokinetic modeling and dose prediction of antibiotics: a scoping review. Antibiotics. 2024;13:1203. 39766593 10.3390/antibiotics13121203 PMC11672403

[129] Minichmayr IK Mizuno T Goswami S . Recent advances addressing the challenges of precision dosing. Clin Pharmacol Ther. 2024;116:527–530. 39087264 10.1002/cpt.3365

[130] Wicha SG Märtson AG Nielsen EI . From therapeutic drug monitoring to model-informed precision dosing for antibiotics. Clin Pharmacol Ther. 2021;109:928–941. 33565627 10.1002/cpt.2202

[131] Launay M Correia P Thiery G . Therapeutic drug monitoring consulting cannot be ruled out by model-informed precision dosing. Ther Drug Monit. 2023;45:706–707. 37226897 10.1097/FTD.0000000000001109

[132] Kantasiripitak W Van Daele R Gijsen M . Software tools for model-informed precision dosing: how well do they satisfy the needs? Front Pharmacol. 2020;11:620. 32457619 10.3389/fphar.2020.00620 PMC7224248

[133] Lin S Cheng X Zhu J . Wearable microneedle-based electrochemical aptamer biosensing for precision dosing of drugs with narrow therapeutic windows. Sci Adv. 2022;8:eabq4539. 36149955 10.1126/sciadv.abq4539 PMC9506728

[134] Rawson TM Gowers SAN Freeman DME . Microneedle biosensors for real-time, minimally invasive drug monitoring of phenoxymethylpenicillin: a first-in-human evaluation in healthy volunteers. Lancet Digit Health. 2019;1:e335-e343. 33323208 10.1016/S2589-7500(19)30131-1

[135] Mishi RD Stokes MA Campbell CA . Real-Time monitoring of antibiotics in the critically ill using biosensors. Antibiotics. 2023;12:1478. 37887179 10.3390/antibiotics12101478 PMC10603738

[136] Parrilla M Detamornrat U Domínguez-Robles J . Wearable microneedle-based array patches for continuous electrochemical monitoring and drug delivery: toward a closed-loop system for methotrexate treatment. ACS Sens. 2023;8:4161–4170. 37856156 10.1021/acssensors.3c01381

[137] Xu X Xu D Zhou X . Implantable photoelectrochemical-therapeutic methotrexate monitoring system with dual-atomic docking strategy. Nat Commun. 2025;16:1747. 39966460 10.1038/s41467-025-57084-2 PMC11836052

[138] Chamorro-Garcia A Gerson J Flatebo C Real-time, seconds-resolved measurements of plasma methotrexate in situ in the living body. ACS Sens. 2023;8:150–157. 36534756 10.1021/acssensors.2c01894

[139] Lin S Yu W Wang B . Noninvasive wearable electroactive pharmaceutical monitoring for personalized therapeutics. Proc Natl Acad Sci USA. 2020;117:19017–19025. 32719130 10.1073/pnas.2009979117 PMC7431025

[140] Gerson J Erdal MK Dauphin-Ducharme P . A high-precision view of intercompartmental drug transport via simultaneous, seconds-resolved, in situ measurements in the vein and brain. Br J Pharmacol. 2024;181:3869–3885. 38877797 10.1111/bph.16471 PMC11890181

[141] Woillard JB Labriffe M Debord J . Tacrolimus exposure prediction using machine learning. Clin Pharmacol Ther. 2021;110:361–369. 33253425 10.1002/cpt.2123

[142] Darwich AS Polasek TM Aronson JK . Model-informed precision dosing: background, requirements, validation, implementation, and forward trajectory of individualizing drug therapy. Annu Rev Pharmacol Toxicol. 2021;61:225–245. 33035445 10.1146/annurev-pharmtox-033020-113257

[143] Poweleit EA Vinks AA Mizuno T. Artificial intelligence and machine learning approaches to facilitate therapeutic drug management and model-informed precision dosing. Ther Drug Monit. 2023;45:143–150. 36750470 10.1097/FTD.0000000000001078 PMC10378651

[144] You W Simalatsar A Widmer N . Personalized drug administrations using support vector machine. BioNanoSci. 2013;3:378–393.

[145] Li QY Tang BH Wu YE . Machine learning: a new approach for dose individualization. Clin Pharmacol Ther. 2024;115:727–744. 37713106 10.1002/cpt.3049

[146] Teplytska O Ernst M Koltermann LM . Machine learning methods for precision dosing in anticancer drug therapy: a scoping review. Clin Pharmacokinet. 2024;63:1221–1237. 39153056 10.1007/s40262-024-01409-9 PMC11449958

[147] Yoon SB Lee JM Jung CW . Machine-learning model to predict the tacrolimus concentration and suggest optimal dose in liver transplantation recipients: a multicenter retrospective cohort study. Sci Rep. 2024;14:19996. 39198694 10.1038/s41598-024-71032-y PMC11358263

[148] Im H Pathania D McFarland PJ . Design and clinical validation of a point-of-care device for the diagnosis of lymphoma via contrast-enhanced microholography and machine learning. Nat Biomed Eng. 2018;2:666–674. 30555750 10.1038/s41551-018-0265-3 PMC6291220

[149] Ahuja K Rather GM Lin Z . Toward point-of-care assessment of patient response: a portable tool for rapidly assessing cancer drug efficacy using multifrequency impedance cytometry and supervised machine learning. Microsyst Nanoeng. 2019;5:34. 31645995 10.1038/s41378-019-0073-2 PMC6799891

[150] Matsumoto T Du L Thoma Y . Simultaneous quantification of multiple drugs by machine learning on electrochemical sensors. In: 2024 IEEE International Symposium on Circuits and Systems (ISCAS). (Singapore, 2024). IEEE. 2024:1–5.

[151] Du L Rodino F Thoma Y . Identification and quantification of multiple drugs by machine learning on electrochemical sensors for therapeutic drug monitoring. IEEE Sensors Lett. 2024;8:1–4.

[152] Marceglia S D'Antrassi P Prenassi M . Point of care research: integrating patient-generated data into electronic health records for clinical trials. AMIA Annu Symp Proc. 2018;2017:1262–1271. 29854195 PMC5977649

[153] Jain S Nehra M Kumar R . Internet of medical things (IoMT)-integrated biosensors for point-of-care testing of infectious diseases. Biosens Bioelectron. 2021;179:113074. 33596516 10.1016/j.bios.2021.113074 PMC7866895

[154] Mujawar MA Gohel H Bhardwaj SK . Nano-enabled biosensing systems for intelligent healthcare: towards COVID-19 management. Mater Today Chem. 2020;17:100306. 32835155 10.1016/j.mtchem.2020.100306 PMC7274574

[155] Park KS Heo H Choi YK. Design and realization of integrated management system for data interoperability between point-of-care testing equipment and hospital information system. Healthc Inform Res. 2013;19:222–228. 24175121 10.4258/hir.2013.19.3.222 PMC3810530

[156] Erasmus R Sahni S El-Sharkawy R. Connectivity strategies in managing a POCT service. EJIFCC. 2021;32:190–194. 34421487 PMC8343042

[157] International Organization for Standardization. ISO 11073-90101:2008(en) Health Informatics – Point-of-care Medical Device Communication – Part 90101: Analytical Instruments – Point-of-care Test. International Organization for Standardization: Geneva, Switzerland; 2008.

[158] Patel NTP Lane MR Williams TK . Hardware and software implementation of POCT1-A for integration of point of care testing in research. J Pathol Inform. 2022;13:100096. 36268088 10.1016/j.jpi.2022.100096 PMC9576979

[159] Kryszyn J Smolik W Wanta D . Comparison of OpenEHR and HL7 FHIR standards. Int J Electron Telecommun. 2023;69:47–52.

[160] Bernardini A Alonzi M Campioni P : IHE: integrating the healthcare enterprise, towards complete integration of healthcare information systems. Rays. 2003;28:83–93. 14509182

[161] Alkarkoukly S Kamal MM Beyan O. Breaking barriers for interoperability: a reference implementation of CSV-FHIR transformation using open-source tools. Stud Health Technol Inform. 2023;302:43–47. 37203606 10.3233/SHTI230061

[162] Weiskopf NG Weng C. Methods and dimensions of electronic health record data quality assessment: enabling reuse for clinical research. J Am Med Inform Assoc. 2013;20:144–151. 22733976 10.1136/amiajnl-2011-000681 PMC3555312

[163] Lewis AE Weiskopf N Abrams ZB . Electronic health record data quality assessment and tools: a systematic review. J Am Med Inform Assoc. 2023;30:1730–1740. 37390812 10.1093/jamia/ocad120 PMC10531113

[164] Jakobsen MI Larsen JR Svensson CK . The significance of sampling time in therapeutic drug monitoring of clozapine. Acta Psychiatr Scand. 2017;135:159–169. 27922183 10.1111/acps.12673

